# Enhancing Flight Connectivity via Synchronization of Arrivals and Departures in Hub Airports with Evolutionary and Swarm-Based Metaheuristics

**DOI:** 10.3390/biomimetics11010006

**Published:** 2025-12-23

**Authors:** Halil Ibrahim Demir, Suraka Dervis

**Affiliations:** 1 Industrial Engineering Department, Sakarya University, 54187 Sakarya, Türkiye; 2 Toyota Motor Europe, 1130 Brussels, Belgium; surakadarwesh@gmail.com

**Keywords:** arrival and departure synchronization, flight scheduling, evolutionary strategies, particle swarm optimization, genetic algorithms

## Abstract

Global air transport has become the dominant mode of long-distance travel, carrying more than four billion passengers in 2019 and projected to exceed 8 billion by 2040. Nevertheless, limited demand and economic inefficiencies often make direct connections unfeasible, forcing many passengers to rely on transfers. In such cases, synchronizing arrivals and departures at hub airports is crucial to minimizing transfer times and maximizing passenger retention. This study investigates the synchronization problem at Istanbul Airport, one of the world’s largest hubs, using metaheuristic optimization. Three algorithms—Genetic Algorithms (GA), Modified Discrete Particle Swarm Optimization (MDPSO), and Evolutionary Strategies (ES)—were applied in parallel to optimize arrival and departure schedules for a major airline. The proposed chromosome-based framework was tested through parameter tuning and validated with statistical analyses, including ANOVA and Games–Howell pairwise comparisons. The results show that MDPSO achieved strong improvements, while ES consistently outperformed both GA and MDPSO, increasing successful passenger transfers by more than 200% compared to the original schedule. These findings demonstrate the effectiveness of evolutionary metaheuristics for large-scale airline scheduling and highlight their potential for improving hub connectivity. This framework is generalizable to other hub airports and airlines, and future research could extend it by integrating hybrid metaheuristics or applying enhanced forecasting methods and more dynamic scheduling approaches.

## 1. Background

Air transport is the leading mode of long-distance travel and reached a peak in 2019 before a sharp decline caused by the COVID-19 pandemic. With the rollout of vaccines and the lifting of restrictions, the sector has entered a recovery phase. Table 1 reports the actual and projected values from 2019 to 2025, showing the rebound in global passenger demand and the expansion of unique city-pair connections. These connections are central to sustaining flows of people, goods, capital, technology, and ideas. These data are drawn from the IATA Global Outlook reports, with 2024 reported as estimates and 2025 as forecasts [1,2].

The International Civil Aviation Organization (ICAO), a United Nations agency, facilitates international cooperation in civil aviation and currently includes 193 member states and over 5000 airlines with ICAO codes. In 2019, prior to the COVID-19 pandemic, global air transport generated USD 876 billion in revenues and carried 4.54 billion passengers across 20,886 unique city-pair connections. The industry employed 70.4 million people within the supply chain, operated a fleet of 29,697 aircraft, and conducted 38.9 million scheduled flights. That year, operations consumed 363 billion liters of fuel, emitted 914 million tons of CO_2_, and incurred labor costs of USD 188 billion, directly employing 2.89 million people. Projections estimate passenger traffic will exceed eight billion by 2040, with the airline market expected to reach USD 16 trillion by 2028. In Europe alone, delays in 2019 totaled 24.2 million minutes, costing USD 2.7 billion in operations and USD 3.2 billion in passenger time.

Table 1 provides a comprehensive overview of the key global airline industry indicators for the period 2019–2025, highlighting both the severe disruptions caused by the COVID-19 pandemic and the subsequent recovery trajectory. The number of unique city pairs, defined as distinct origin–destination airport combinations served by at least one scheduled commercial flight within a given year, serves as a critical measure of global network connectivity. Each city pair is counted only once, irrespective of how many airlines or frequencies operate on that route.

As shown in Table 1, worldwide connectivity sharply contracted from 20,886 unique city pairs in 2019 to 16,218 in 2020 due to the collapse in international mobility. Despite a slight improvement in 2021, connectivity remained far below pre-pandemic levels. A notable recovery began in 2022, and by 2023 the network exceeded its pre-crisis breadth, reaching 21,736 city pairs. Passenger demand and revenue streams exhibit a similar pattern, with a dramatic decline in 2020 followed by gradual recovery through 2023 and positive projections for 2024–2025. Financial indicators also reflect this cycle: while the industry recorded a historic net loss of USD –137.7 billion in 2020, profitability returned by 2023.

Overall, Table 1 illustrates the resilience of the global air transport system. The evolution of passengers, revenues, aircraft departures, and especially unique city pairs demonstrates how the industry first contracted under pandemic pressures and then underwent a steady restoration of operational scale and network connectivity.

As Table 1 indicates, the years 2020–2022 were heavily impacted by the pandemic, while global recovery has only been observed since 2023 [1,2,3,4,5].

Air travel in Türkiye has grown significantly faster than the global average over the past two decades, driven by major investments and an expansion of airports from 39 in 2003 to 60 in 2024, with eight more planned or under construction. According to Table 2, Domestic passenger traffic rose from ~9 million in 2003 to ~95 million in 2024. Türkiye’s strategic East–West and North–South location, coupled with a tourism boom, contributed to this growth, with tourist arrivals increasing from 16.5 million (USD 13.2 billion revenue) in 2003 to over 52 million (USD 61 billion) in 2024. Rising international passenger volumes—from ~25 million in 2003 to over 134 million in 2024—reflect both outbound travel by Turkish citizens and Türkiye’s role as a global transfer hub, particularly via the newly opened Istanbul Airport. Pandemic effects persisted through 2020–2022. Türkiye rose from 18th in the world for total passengers in 2003 to 6th globally and 2nd in Europe by 2024. Air transport revenues grew from USD 2.2 billion in 2003 to USD 29.73 billion in 2023 [6,7,8,9].

The literature on airport and airline operations covers numerous problems, including flight scheduling, fleet assignment, aircraft routing, crew scheduling, revenue and fuel management, irregular operations, gate assignment, boarding strategies, hub-and-spoke network planning, demand and maintenance planning, and arrival/departure/runway scheduling. While flight scheduling and network optimization are well-studied, arrival and departure synchronization remain underexplored. The present study seeks to fill this research gap.

Despite the breadth of research on airline operations, the specific problem of airline or hub-level arrival–departure synchronization remains insufficiently explored in the literature. Our review identified only two studies that address problems closely related to synchronization dynamics. The first is Çiftçi and Özkır [10], who optimized flight connection times within airline bank structures using Simulated Annealing and Tabu Search, demonstrating that metaheuristic approaches can effectively reduce passenger misconnections and improve temporal coordination. The second is Seredyński et al. [11], who examined the impact of timetable synchronization on hub connectivity for European carriers, showing that coordinated scheduling enhances network accessibility. However, neither study addresses integrated arrival–departure synchronization at the hub level using contemporary evolutionary or swarm-based metaheuristic methods. Accordingly, the present study constitutes the first attempt to apply Genetic Algorithms (GA), Modified Discrete Particle Swarm Optimization (MDPSO), and Evolutionary Strategies (ES) to this underexplored synchronization problem, offering a novel methodological contribution to airline hub operations.

Recent studies have further advanced research on arrival–departure scheduling, synchronization, and hub coordination, offering insights directly relevant to the scope of the present study. Ma et al. [12] developed a mixed-integer programming model for the Aircraft Sequencing Problem (ASP), jointly optimizing runway assignments and landing, take-off, and crossing times under realistic operational constraints. Their results show that integrated sequencing and runway optimization substantially reduce delays, reinforcing the importance of coordinated arrival–departure management. Similarly, Zhou et al. [13] reframed flight arrival slot allocation as a language-modeling task, demonstrating that large language models (LLMs) can generate conflict-free arrival sequences and effectively minimize temporal conflicts—an emerging AI-driven approach that complements optimization-based synchronization.

Wei et al. [14] proposed a multi-objective fuzzy model for multi-type aircraft scheduling and, using an NSGA-II–based heuristic, achieved significant reductions in idle time and fleet size, thereby contributing to operational efficiency in hub environments. Yang et al. [15] introduced a Mixed-Integer Nonlinear Programming (MINLP) framework for multi-hub flight scheduling, enhanced by a Selective Simulated Annealing algorithm, and demonstrated notable improvements in connectivity using real-world Air China data—findings directly relevant to hub-based arrival–departure optimization.

Additionally, hub-and-spoke network studies, such as Sindhwani et al. [16], highlight the role of regional hub selection in enhancing accessibility and resilience, whereas Yu et al. [17] present a multiple-allocation hub design model (HURO) that significantly improves profitability through submodular optimization with approximation guarantees. Broader perspectives on hub management and slot coordination include Rupp et al. [18], who optimized consolidation processes in multimodal hubs; Brinton et al. [19], who developed the GRBS algorithm for detailed flight slot allocation; Yang et al. [20], who examined stochastic terminal scheduling; and Schultz et al. [21], who introduced a long-range air traffic flow management concept for the Asia–Pacific region. Despite these advances, the synchronization and joint optimization of flight arrivals and departures at hub airports—particularly using the solution approach employed in this study—remain only partially explored.

For clarity, this study focuses only on the most relevant airline operations: flight scheduling, fleet assignment, and arrival/departure synchronization. Other operations are not detailed.

This study is structured as follows: Section 2 reviews relevant literature on airport and airline operations, focusing on flight scheduling, fleet assignment, and synchronization. Section 3 defines the problem, Section 4 explains İstanbul Airport case, Section 5 presents potential solutions, Section 6 discusses results, and Section 7 provides conclusions.

## 2. Literature Survey

Despite extensive research on arrival/departure scheduling, flight planning, and hub-and-network optimization, the synchronization of arrivals and departures remains underexplored, a gap that is addressed in the present study.

For further reference, key studies on airport and airline operations include air traffic management [10,22,23,24,25,26,27], aircraft routing [28,29], arrival/departure scheduling [12,13,14,15,20,30,31], ground operations [32,33], and hub-and-spoke network planning [16,17,34,35,36].

To maintain analytical focus, the literature review concentrates exclusively on the airline operations most relevant to the scope of this study—namely flight scheduling, fleet assignment, and the synchronization of arrivals and departures—while other operational processes fall outside the present discussion. Flight scheduling, the initial stage in airline planning, involves creating timetables; determining origins, destinations, and arrival/departure times; and frequency planning. This stage significantly affects profitability by influencing revenue and operating costs. It requires consideration of market demand, available aircraft, crew availability, and regulatory constraints [37,38,39].

Slot allocation and scheduling research largely seeks to balance demand with limited airport and airspace capacity, yet studies diverge in how they account for delays, uncertainty, and airline profitability. Traditional approaches often prioritize airline objectives while overlooking primary sources of delay. To address this gap, data-driven and stochastic models have been developed to capture schedule deviations [40,41], routing and flow management under uncertainty [42,43,44], and predictive tools for delay and cancellation risk [45]. Complementary approaches, such as flight retiming, offer cost-efficient improvements in service quality [46]. Collectively, these studies emphasize the persistent challenge of congestion, variability, and infrastructure constraints in ensuring reliable operations.

The literature widely addresses integrated flight scheduling and fleet assignment. Pita et al. [47] studied this problem under airport congestion—the leading cause of costly delays, estimated at $32.9 billion for the U.S. in 2007—and proposed a mixed-integer linear optimization model that incorporates both aircraft and passenger delay costs. Although this estimate refers to the U.S. system in 2007, it is cited here not for regional comparison but to illustrate the magnitude of delay-related costs in large hub networks. The figure highlights that inadequate arrival–departure coordination can generate substantial economic losses through extended ground waiting times, missed connections, and propagated delays. This motivates the emphasis on synchronization in the present study, where reducing transfer times and minimizing airport waiting costs are essential for improving hub efficiency.

Following scheduling, fleet assignment emerges as the next critical driver of profitability, requiring alignment of aircraft capacity with uncertain demand while managing operational cost–revenue trade-offs [38,39]. Research has increasingly treated scheduling and fleet assignment as integrated problems, motivated by the significant economic impact of delays Pita et al. [47] and the need to account for demand elasticity and labor constraints [38,39]. Recent contributions have refined these integrated models by incorporating more explicit demand–supply dynamics [48].

Overall, the literature highlights a shift from the isolated optimization of scheduling or fleet assignment toward integrated, uncertainty-aware models that better capture the complex trade-offs among efficiency, reliability, and profitability in modern airline operations.

Research on airline operations highlights the interdependence of fleet assignment, aircraft routing, crew scheduling, and revenue management, where optimization at each stage yields efficiency gains and cost savings [37,38,39]. Recent studies have advanced this literature by incorporating environmental considerations [49], heuristic approaches to fleet sizing [50], and passenger choice in integrated scheduling models [51]. Beyond aviation, synchronization has been explored in rail, bus, and delivery networks [52,53,54,55,56,57,58,59] with the closest analogies to air transport found in hub-based coordination.

The widespread adoption of hub-and-spoke (HS) systems following deregulation in the U.S. and later in Europe and other regions transformed airline operations, simultaneously expanding consumer access and enhancing carrier efficiency [60,61]. Collectively, this body of work underscores how synchronization, integration, and network design remain central to balancing efficiency, profitability, and service quality in modern air transport.

The dominance of hub-and-spoke networks complicates the assessment of airport competitiveness and accessibility, as size-based measures tend to overstate hub performance while undervaluing smaller airports connected to major nodes [62]. To address this limitation, a range of refined connectivity indices has been developed. For example, the Continuous Connectivity Index by Lee et al. [61] captures airline schedule coordination across global markets, while the Weighted Connectivity Score by Seredyński et al. [11] and related measures by Rietveld and Brons [63] emphasize the role of timetable synchronization in improving effective connectivity. Similarly, Danesi [64] proposed multiple indices to evaluate hub coordination, and Paleari et al. [65] compared accessibility and travel times across major U.S., European, and Chinese networks. Collectively, these studies underscore the importance of advanced, quality-sensitive indices that better capture accessibility and network performance than traditional size-based measures.

In addition to the extensive research on airline planning and synchronization, the present study employs several well-established metaheuristic optimization techniques to address the computational complexity of the proposed model. Genetic Algorithms (GA), originally introduced by Holland, provide strong global search capabilities by simultaneously evaluating a population of candidate solutions, enabling effective exploration of large combinatorial search spaces. The algorithmic structure used in this study follows the flowchart presented in Section 5, and GA have consistently been shown to yield near-optimal results within reasonable computation times [66].

Complementing GA, Particle Swarm Optimization (PSO) and its variant, the Modified Discrete PSO (MDPSO)—whose procedural framework is illustrated in Section 5—offer an alternative population-based search mechanism in which individuals update their positions through directed stochastic movements rather than generational selection [67].

Finally, Evolutionary Strategies (ES), originally developed by Rechenberg and Schwefel and depicted in Section 5, represent another mutation-driven evolutionary paradigm governed by the (µ, λ) selection mechanism, emphasizing incremental local refinement over crossover operations [67]. Collectively, these three metaheuristic approaches constitute the core solution methodology adopted in the present study due to their robustness and suitability for large-scale, nonlinear, and combinatorial optimization problems.

## 3. Problem Definition

Most air passengers rely on transfers to reach their destinations. To minimize passenger loss and maximize retention during transfers, airlines must optimally synchronize arrival and departure schedules. This study seeks to achieve such synchronization using meta-heuristic approaches, thereby reducing missed connections and improving passenger flow.

Global air transport continues to expand, driven by technological advances, rising living standards, population growth, and increasing international and domestic routes. With over 38 million annual flights and more than 5000 airlines worldwide, synchronization and optimization offer both substantial cost savings for carriers and enhanced comfort and affordability for passengers.

Direct flights are often infeasible due to economic constraints, making connections essential across domestic–domestic, international–international, and cross-border journeys. Since travelers prioritize short travel times, low costs, and comfort, airlines with minimal transfer waiting times gain a competitive advantage.

Airports function as hubs, which vary by regional model. The U.S. model primarily connects medium-haul flights, the European model links medium- to long-haul services, and Gulf and Asian hubs also interconnect long-haul flights. Major hubs include JFK, LAX, and ATL in the U.S.; LHR, IST, FRA, and CDG in Europe; DXB and DOH in the Gulf; and DEL, CGK, PEK, and HND in Asia.

**Model Assumptions**

The following assumptions and constraints were adopted based on prior literature [34,68] and further refined to enhance model realism and clarity, while maintaining computational tractability and focusing on the core problem of maximizing transfer passenger retention through arrival–departure synchronization.

a. Passenger transfer behavior

Passenger retention is assumed to depend primarily on transfer waiting time, as summarized in Table 3. Extremely short transfers (0–1 h) are assumed to result in passenger loss due to infeasible connection times, while intermediate transfer windows (1–3 h) ensure full retention. Excessively long waiting times (e.g., above 10 h) are assumed to lead to complete transfer abandonment. Additional factors such as individual mobility limitations, refreshment needs, or discretionary passenger activities are not explicitly modeled and are implicitly reflected in the transfer time preference structure.

b. Transfer buffer homogeneity

A uniform transfer buffer is assumed for all passengers, regardless of special assistance needs (e.g., reduced mobility passengers) or discretionary behaviors (e.g., refreshments or lounge usage). This simplification allows the model to focus on network-level synchronization effects rather than passenger-specific service heterogeneity.

c. Aircraft operational reliability

Aircraft are assumed to operate without technical malfunctions or unscheduled maintenance requirements. This constraint isolates the impact of timetable coordination from irregular operational disruptions.

d. Weather and exogenous disruptions

Weather-related delays, air traffic control disruptions, and other exogenous operational disturbances are not explicitly considered. While these factors affect real-world operations, they are excluded to evaluate baseline synchronization performance under nominal operating conditions, consistent with an initial-stage optimization framework.

e. Inter-flight delay propagation

Delay propagation between consecutive flights is not modeled explicitly. Arrival and departure times are treated as planned schedule times, enabling the assessment of synchronization quality without compounding stochastic delay effects.

f. Demand stability

Passenger demand is assumed to be static and deterministic over the planning horizon. Dynamic demand variations and real-time booking adjustments are beyond the scope of the present study.

g. Pricing and fare effects

Ticket prices, fare classes, and revenue management considerations are excluded. The objective focuses exclusively on passenger transfer feasibility rather than revenue maximization.

h. Fleet and aircraft constraints

Aircraft type restrictions, crew constraints, and fleet rotation dependencies are not imposed. This allows the model to concentrate on arrival–departure temporal alignment at the hub level.

This study aims to minimize passenger loss in connecting flights by synchronizing arrivals and departures. Inputs include origin and destination points relative to the hub and the potential passenger flows between them. 

**Constraints**

The optimization model is subject to the following constraints, which are designed to ensure operational feasibility and consistency with the observed flight schedule characteristics reported in Table 4 and Table 5.

a. Network structure constraint

Simultaneous scheduling shall allow arrivals from up to a origin airports to the hub airport and departures from the hub airport to up to d destination airports. This constraint preserves the predefined hub-and-spoke network structure.

b. Weekly scheduling horizon constraint

All flights shall be scheduled within a weekly planning horizon, commencing at 00:00 on Monday and concluding at 23:55 on Sunday. This temporal framework is fully consistent with the weekly flight frequencies reported in Table 5 and ensures that all scheduled arrivals and departures are allocated within the same weekly cycle.

c. Demand realization constraint

The scheduling process shall aim to maximize the realization of customer potential for each origin–destination pair, as specified in Table 4. Passenger transfer feasibility is evaluated based on the arrival–departure time alignment within the defined transfer time thresholds.

d. Flight frequency constraint

As detailed in Table 5, a predetermined number of weekly flights shall be maintained between the hub airport and each destination, in both arrival and departure directions. These weekly frequencies are treated as fixed input parameters derived from the observed schedule and are imposed as hard constraints to ensure consistency with real-world airline operations.

**Chromosome Representation:** In the proposed model, each arrival and departure chromosome is composed of two axes. The X-axis denotes the sequence of scheduled arrivals or departures, while the Y-axis represents the day and discrete five-minute time slots allocated to these operations. For both chromosomes, the X-axis accommodates a maximum of “a” arrivals and “d” departures. Correspondingly, the Y-axis encompasses 2016 distinct five-minute intervals across a weekly schedule, as illustrated in Table 6 and Table 7. This representation enables precise modeling of scheduling constraints and facilitates the optimization of arrival–departure synchronizations.

## 4. Istanbul Airport Case

**Figure 1:**
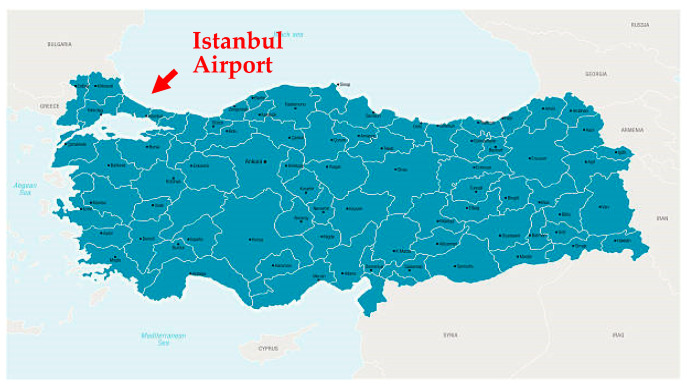
Location of Istanbul Airport.

**Figure 2:**
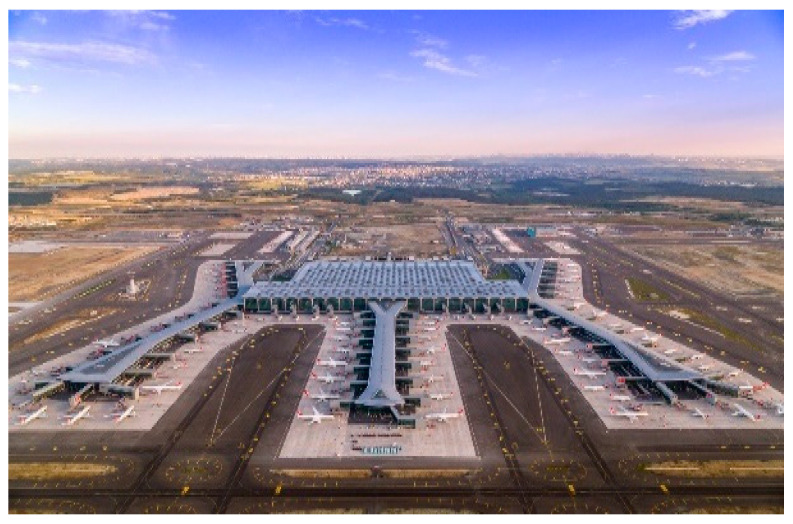
Istanbul Airport.

**Figure 3:**
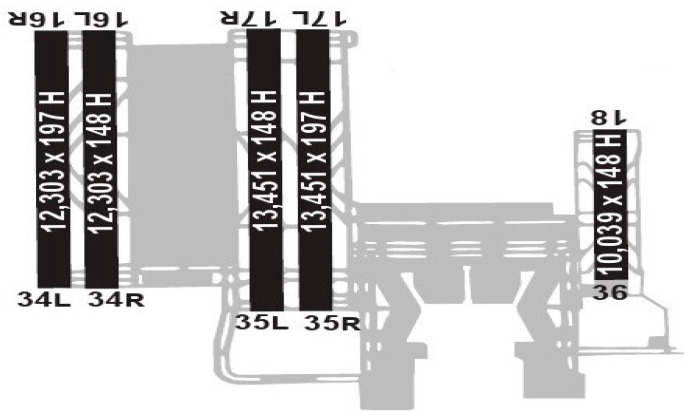
Airport Runways.

This optimization problem can be applied to any major hub airport; however, the present study focuses specifically on Istanbul Airport. Commissioned in 2018, Istanbul Airport serves as the principal international gateway for both Istanbul and Türkiye. This airport’s strategic geographic location renders it a critical transit hub connecting Eurasia, Africa, and the Americas. Situated on the European side of the city (Figure 1, Figure 2 and Figure 3), the airport is designated by IATA code IST and ICAO code LTFM.

The limitations of the previous Istanbul Atatürk Airport, particularly insufficient space for additional runways, constrained the capacity for charter flights, cargo operations, and other services, while congestion increasingly impeded operations. These challenges necessitated the development of a new airport. Construction began in 2015, and operations commenced in 2018. Comprehensive statistics regarding Istanbul Airport’s capacity and operations are presented in Table 8 and Table 9 [69].

As previously noted, Istanbul Airport occupies a strategic position within global air transport networks. In this study, the problem in question was specifically examined for Istanbul Airport, which serves as a major international hub. At the time of analysis, the airport accommodated 401 departure and 401 arrival points for a leading Turkish airline. Operational constraints allowed a maximum of seven arrivals (a) and seven departures (d) to be scheduled within the same five-minute interval. This operational limit underpins the synchronization and optimization model proposed in this study, ensuring realistic constraints are incorporated into the scheduling framework to enhance transfer efficiency and passenger throughput.

## 5. Solution Methods

**Figure 4:**
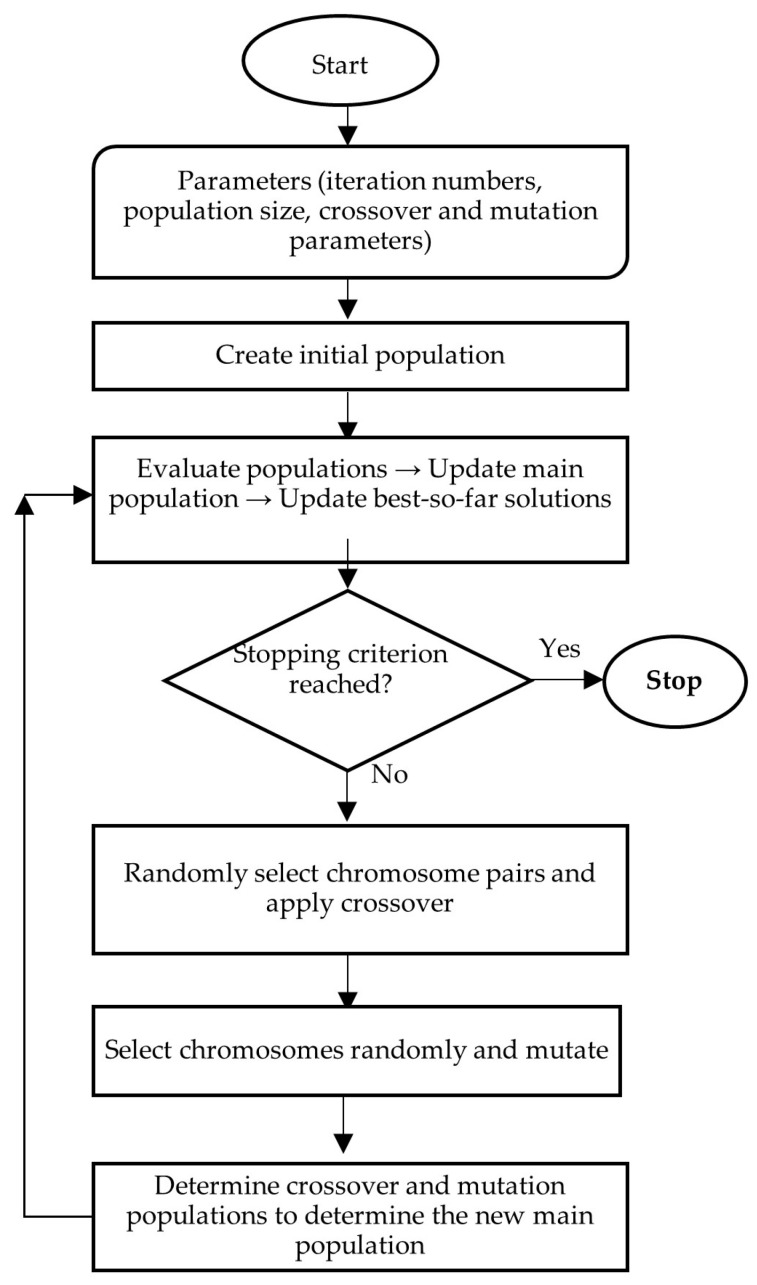
GA flow chart.

**Figure 5:**
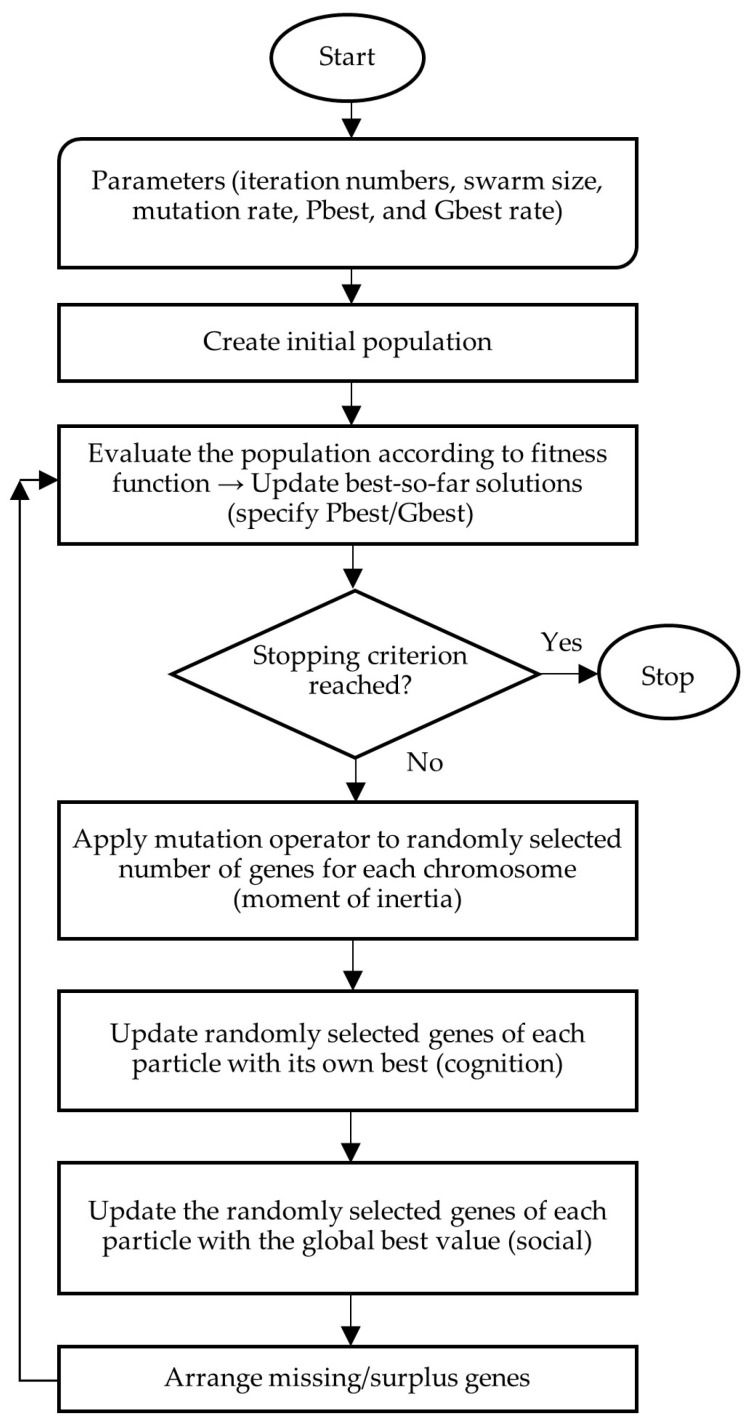
MDPSO flow chart.

**Figure 6:**
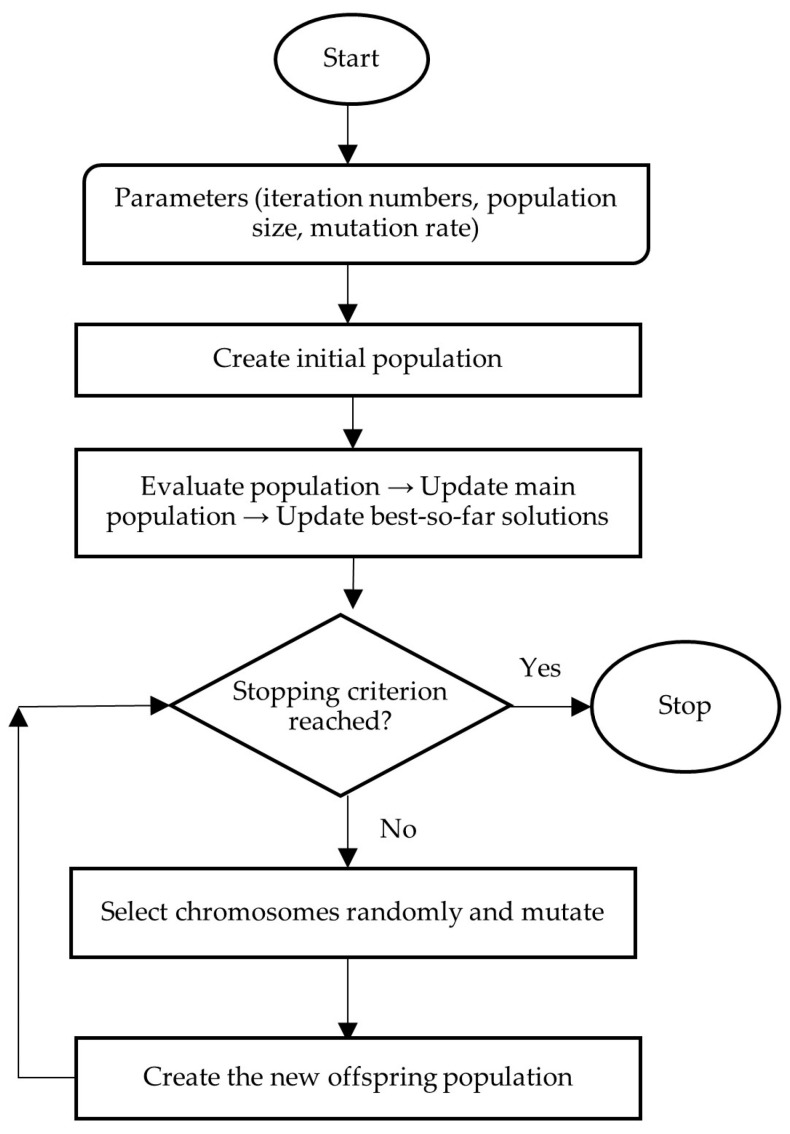
ES flow chart.

This study compares the airline’s original arrival–departure plan (OP) with results from three metaheuristic approaches: Genetic Algorithms (GA), Modified Discrete Particle Swarm Optimization (MDPSO), and Evolutionary Strategies (ES), with flow diagrams provided in Figure 4, Figure 5 and Figure 6.

The model uses two two-dimensional chromosomes—one for arrivals, one for departures—where the Y-axis denotes planned time slots and the X-axis specifies assigned flights. Arrival solutions are applied analogously to departures. Distinct from existing work, the algorithms are executed in parallel for both populations, enabling concurrent optimization of arrival and departure schedules to enhance synchronization and operational efficiency.

### 5.1. Mathematical Model for Synchronization of Arrivals and Departures

This section presents a formal mathematical model aimed at maximizing passenger transfers by optimizing the synchronization of arrivals and departures, while incorporating operational constraints, inter–origin–destination demand, runway capacities, and connecting time differences. Given the complexity of an airline network involving approximately 401 arrival points and 401 destination points, the problem becomes computationally prohibitive for exact optimization. Therefore, a reduced-size illustrative instance consisting of five arrival and five destination points is considered to ensure tractability. Flight demand and feasible scheduling windows for these selected points are incorporated, and the model is solved using the LINGO 20.0.12 software, with the assumed demand matrix reported in Table 10. This illustrative formulation is used to clearly demonstrate the structure of the objective function, decision variables, and constraints, while the full-scale problem is addressed using the proposed metaheuristic solution approaches.
$Max\;Z=\sum_{i=1}^{5}.\sum_{j=1}^{5}{PasX}_{ij}$
(1)*Subject to*

${TimeA}_{i}=$      $\sum_{p=1}^{5}A_{ip}*T_{p}$*i =* 1, …, 5(2)${TimeD}_{j}=\sum_{p=1}^{5}D_{jp}*T_{p}$*j =* 1, …, 5(3)${TotalA}_{i}=\sum_{p=1}^{5}A_{ip}$*i =* 1, …, 5(4)${TotalD}_{j}=\sum_{p=1}^{5}D_{jp}$*j =* 1, …, 5(5)${RunA}_{p}=\sum_{i=1}^{5}A_{ip}$*p =* 1, …, 5(6)${RunD}_{p}=\sum_{i=1}^{5}D_{jp}$*p =* 1, …, 5(7)${DemX}_{ij}=Demand\;from\;arrival\;point\;i\;to\;departure\;point\;j$(8)${TimeDifX}_{ij}=\;$*Max {*${TimeD}_{j}-{TimeA}_{i}$, 0*}**i =* 1, …, 5     *j =* 1, …, 5     *i ≠ j*(9)${PasX}_{ij}$*=*$\sum_{i=1}^{5}.\sum_{j=1}^{5}R_{ij}*{DemX}_{ij}\;$*i =* 1, …, 5     *j =* 1, …, 5     *i ≠ j*(10)${TotalA}_{i}\;=\;1$                  ${TotalD}_{j}=\;1$*i =* 1, …, 5     *j =* 1, …, 5(11), (12)${RunA}_{p}\leq 2$               ${RunD}_{p}\;\leq 2$*p =* 1, …, 5(13), (14)$A_{ip}\;=\;0\;or\;1$                   $D_{jp}\;=\;0\;or\;1$*i =* 1, …, 5     *j =* 1, …, 5     *p =* 1, …, 5(15), (16)All *variables ≥* 0,       ${RunA}_{p},\;{RunD}_{p}$*,*
${DemX}_{ij},\;{TimeA}_{i},\;{TimeD}_{j}$
*are integer variables*

**Model Explanation:**

(1) Objective function: Maximizes the total number of passengers successfully transferred.

(2), (3) Define arrival and departure times based on scheduled time slots *T_p_* and binary assignment variables.

(4), (5) Ensure each arrival and departure point has exactly one flight.

(6), (7) Ensure runway capacity limits are not exceeded for arrivals and departures.

(8) Demand between arrival and departure points.

(9) Time difference between arrivals and departures, ensuring non-negative values.

(10) Passengers gained from transfers, based on demand and time-dependent transfer ratio Rij.

(11), (12) Ensure exactly one arrival/departure for each point.

(13), (14) Limit simultaneous arrivals/departures to runway capacity.

(15), (16) Binary variables indicating if a flight is scheduled from/to a point at a given time.

**Variable Definitions:**
*i =* 1, …, 5 :Arrival points (1, …, 5)*j =* 1, …, 5 :Departure points (1, …, 5)

$T_{p}$

:(0–5, 5–10, 10–15, 15–20, 20–25) = Flight time slots (T1, T2, T3, T4, T5) “In the LINGO model, the time slot 0–5 is assumed to correspond to an arrival or departure occurring at time 5.”$A_{ip}$*=* 0 *or* 1:Binary variable for arrival from point *i* at time p$D_{jp}$*=* 0 *or* 1:Binary variable for departure to point *j* at time *p*${RunA}_{p}$ ≤2:Number of arrivals at time *p* should be less than or equal to 2${RunD}_{p}$ ≤2:Number of departures at time *p* should be less than or equal to 2${TotalA}_{i}\;$*=* 1 :There is only 1 flight from every arrival point${TotalD}_{j\;}$*=* 1:There is only 1 flight to every departure point

${DemX}_{ij}$

:Passenger demand from *i* to *j*

${TimeDifX}_{ij}$

:Time difference between arrival *i* and departure *j* (Negative values = zero)
*R_ij_*
:

$\mathrm{Transfer}\;\mathrm{passenger}\;\mathrm{ratio}\;\mathrm{from}\;i\;\mathrm{to}\;j\;\mathrm{depending}\;\mathrm{on}\;\mathrm{time}\;\mathrm{difference}\;{TimeDifX}_{ij}$



${PasX}_{ij}$

:Number of passengers transferred from *i* to *j*


### 5.2. Genetic Algorithms (GA)

In this study, the Genetic Algorithm is employed as an iterative search mechanism designed to improve arrival and departure schedules under hub-airport operational constraints. The implementation begins with the construction of feasible initial chromosomes representing complete flight assignments. Problem-specific operators are applied to explore alternative configurations while preserving feasibility. Candidate solutions are evaluated using the objective function, and improved schedules are retained until convergence behavior indicates no further significant improvement.

### 5.3. Modified Discrete Particle Swarm Optimization (MDPSO)

The Modified Discrete Particle Swarm Optimization (MDPSO) approach is employed as a population-based search method that enables rapid iterative improvement of arrival and departure schedules. In this study, each particle represents a feasible timetable configuration encoded with integer-valued genes, and the algorithm is applied simultaneously to both arrival and departure populations. Particle movements within the solution space are guided by both individual historical performance and the globally best solutions identified by the swarm. After each update, feasibility restoration procedures are applied to ensure full flight coverage and compliance with predefined time-window constraints, allowing coordinated improvement across both timetable structures through parallel evaluation.

The iterative process proceeds as follows. First, inertia genes are randomly selected from each individual and reassigned through a mixing operation. Next, during the cognition step, each particle moves toward its personal best solution by replacing a number of genes—determined by the mutation rate—with corresponding genes from its best-known configuration. In the subsequent social step, particles move toward the global best solution found by the swarm, again replacing genes according to the mutation rate. Following these updates, any surplus flights are randomly removed and replaced with missing flights to restore feasibility, thereby completing one iteration of the algorithm. This procedure is repeated iteratively and applied in parallel to both arrival and departure populations.

### 5.4. Evolutionary Strategies (ES)

Evolutionary Strategies (ES) are employed as a mutation-driven optimization mechanism aimed at refining feasible arrival and departure schedules through controlled and systematic adjustments. In this study, the ES framework incorporates several algorithmic parameters alongside the defined problem constraints, including the iteration number, population size, mutation rate, and the proportion of individuals subject to mutation. The initial population is constructed to fully satisfy all feasibility requirements, after which candidate solutions are iteratively evaluated using the objective function.

During each iteration, a subset of individuals is selected according to the specified mutation proportion and subjected to random alterations at predefined gene positions, while structural feasibility is preserved. Flights corresponding to mutated genes are reassigned randomly within the same positions, ensuring consistency in the scheduling structure. Following mutation, updated solutions are evaluated against the objective function, and superior individuals are retained through the selection process. These procedures are executed in parallel for both arrival and departure chromosomes and repeated until the predefined stopping criterion is satisfied.

## 6. Results and Discussion

### 6.1. Mathematical Model Results

The small-scale problem was solved using LINGO 20.0.12, yielding a total of 149 acquired passengers. The corresponding results are presented in Table 11, Table 12 and Table 13.

As detailed in Section 4, three algorithms were implemented and compared against the airline’s original schedule. The original plan (OP) of the airline company yields 65,659 transfer passengers, and this value is compared with the transfer passenger gains obtained using the three metaheuristic methods. Computations were performed in Python 3.8 on a system with an Intel Core i7-5500U processor (2.40 GHz, 8 GB RAM) running Windows 10 (64-bit). The results when using Genetic Algorithms (GA) with one-point, two-point, and three-point crossovers are presented in Table 14. A one-way ANOVA (unequal variances) revealed no significant differences across the crossover methods for best or average outcomes (best: F = 2.43, *p* = 0.157; average: F = 0.21, *p* = 0.814). Games–Howell pairwise comparisons confirmed this result, as all intervals contained zero. However, in terms of both average best and average values, the three-point crossover consistently outperformed others. Consequently, subsequent comparisons with alternative metaheuristics employed GA with a three-point crossover. The detailed results are shown in Table 15.

Based on extensive experiments, MDPSO and ES consistently outperformed other methods. A parameter sensitivity analysis was subsequently conducted, testing combinations of 5, 10, and 15 chromosomes with mutation rates of 100, 300, and 500 genes. To ensure fairness, the program executed 100 iterations for 5 chromosomes, 50 for 10, and 34 for 15. As shown in Table 16, the 5-chromosome–300-gene configuration yielded the best performance. This optimal configuration was then applied to run the MDPSO and ES algorithms for 200 iterations. To ensure reproducibility, seed logic was implemented, and experiments were repeated with three seeds: 8401, 8402, and 8403.

**Figure 7:**
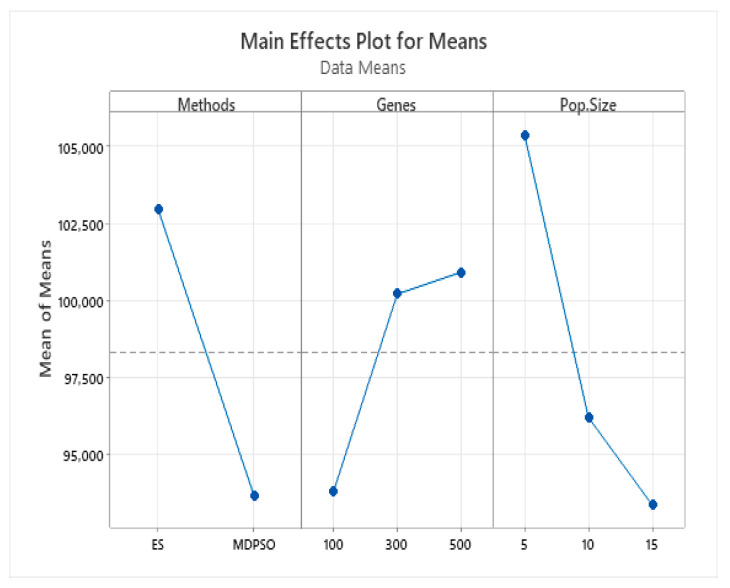
Taguchi experimental results of main effects plot for means. The horizontal dashed line represents the overall mean of the response variable across all factor levels.

We applied a Taguchi experimental design (L18(2^1^)(3^2^)) to the above data, examining three factors: algorithm type (ES or MDPSO), mutation gene count (100, 300, or 500), and population size (5, 10, or 15). Using the Minitab 21.4.1 software, the analysis revealed that the ES algorithm consistently outperformed MDPSO. Mutation rates of 300 and 500 genes yielded strong results, with 500 genes performing best overall. A population size of 5 was identified as optimal (Figure 7).

**Figure 8:**
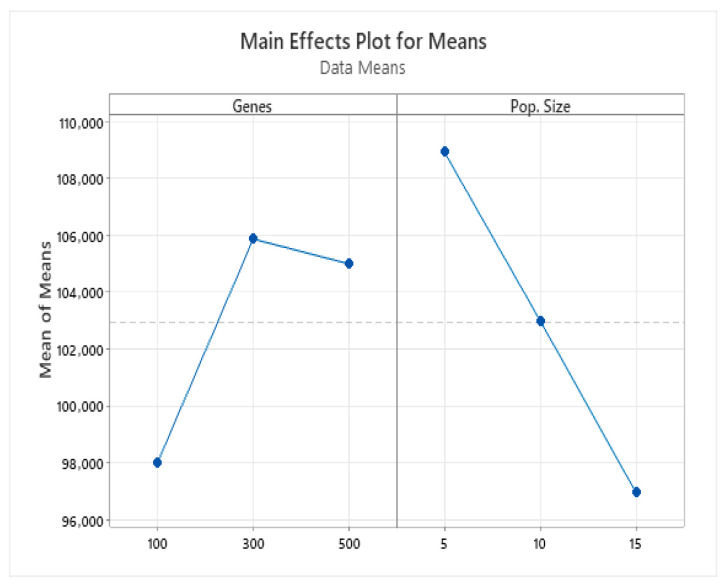
ES Algorithm results of main effects plot for means. The horizontal dashed line represents the overall mean of the response variable across all factor levels.

Subsequently, a Taguchi experimental design (L9(3^2^)) was applied exclusively to the ES algorithm using nine observations. The mutation gene counts mirrored those in the previous analysis, and the population sizes were set to 5, 10, and 15. The results (Figure 8) indicate that 300 mutated genes yielded optimal performance for ES, with a population size of 5. For a comparative analysis across algorithms, we standardized the iteration settings: GA employed a population size of 10 with 100 iterations to balance crossover and mutation, while ES and MDPSO, both optimized at a population size of 5, were run for 200 iterations under identical parameter settings to ensure fairness.

### 6.2. Genetic Algorithm Performance

**Figure 9:**
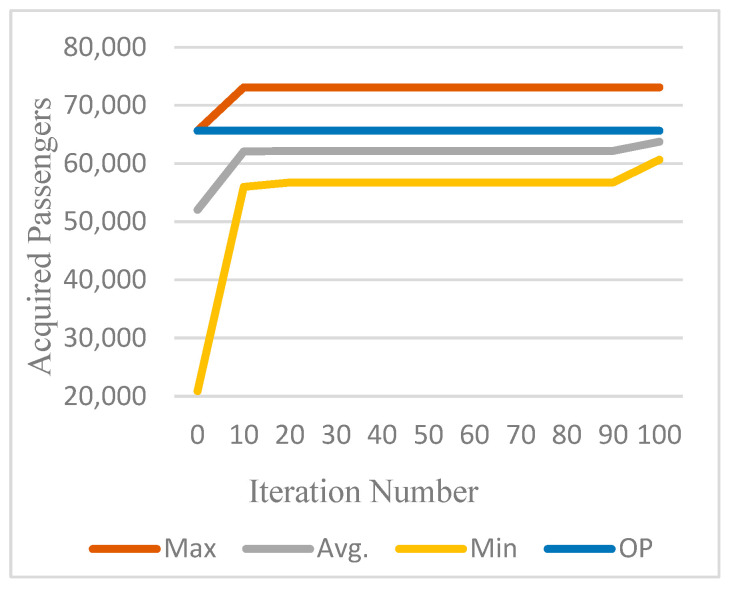
Comparison of best, average, and worst results across 100 iterations for GA seed-1.

The GA program was executed with three distinct seeds, yielding an average CPU time of approximately 90 min. Table 17 presents the results recorded for every 20 iterations. Notably, in the final iteration, seed-1 achieved the highest performance, securing 73,107 passengers and an average of 65,327 passengers. These findings are illustrated in Figure 9.

### 6.3. Modified Discrete Particle Swarm Optimization Performance

The MDPSO algorithm was executed with three different seeds, averaging approximately 90 min of CPU time. Table 18 presents the results for each seed at 40-iteration intervals. In the final iteration, seed-3 achieved the highest outcome with 118,092 passengers and the greatest average of 108,236 passengers.

**Figure 10:**
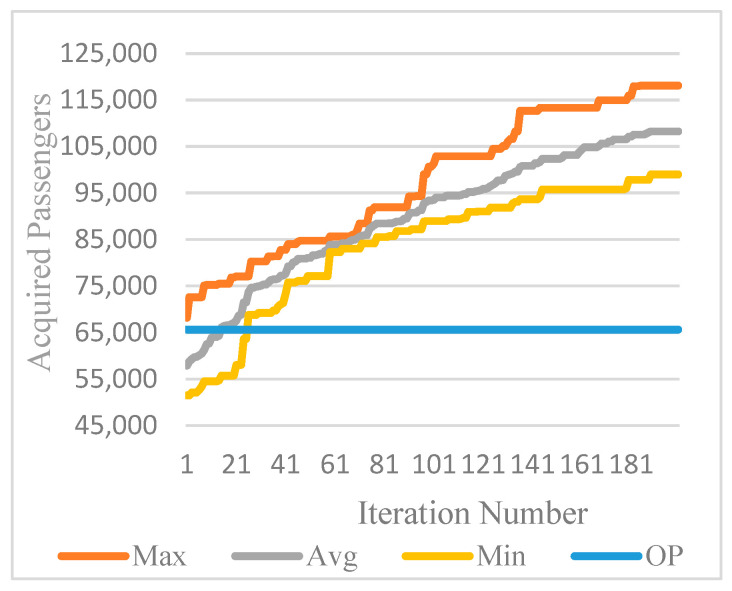
Comparison of best, average, and worst outcomes across 200 iterations for MDPSO seed-3.

Figure 10 compares the best, average, worst, and original plan (OP) passenger numbers over 200 iterations for seed-3, showing that average performance surpasses the original plan after iteration 17.

### 6.4. Evolutionary Strategy Performance

The ES program was executed for three seeds, with an average CPU time of 19 min. Table 19 presents results at every 40 iterations. In the final iteration, seed-2 achieved the highest performance, yielding 147,596 passengers and the highest average of 146,019 passengers.

**Figure 11:**
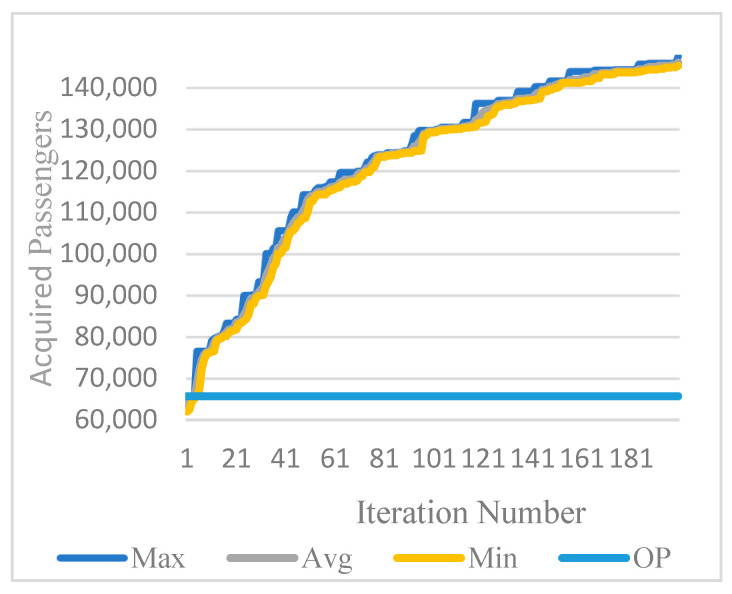
Best, average, and worst results over 200 iterations for ES (seed 2).

**Figure 12:**
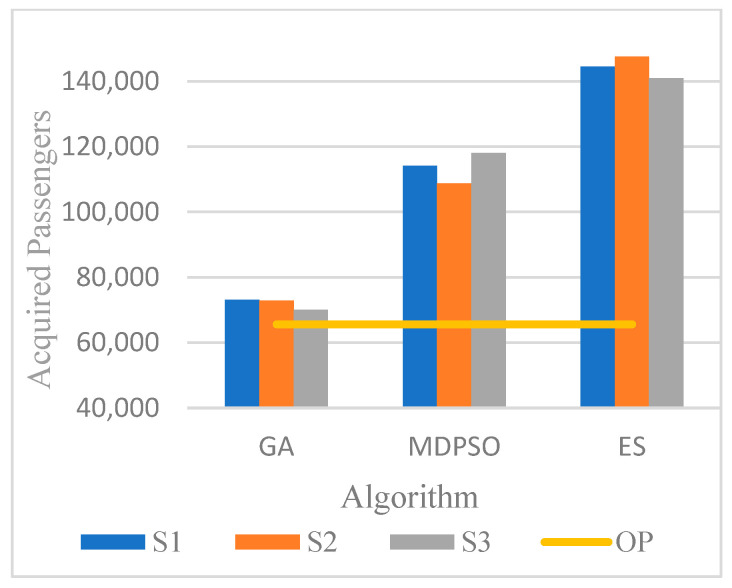
Comparative analysis of the best outcomes across all algorithms and seeds.

Figure 11 compares the best, average, worst, and original plan (OP) passenger numbers over 200 iterations for seed-2. Here, after iteration 7, the average results improve beyond those of the original plan. Figure 12 compares all algorithms with the original plan. Here, ES offers the best performance, and MDPSO also appears to be a promising algorithm.

**Figure 13:**
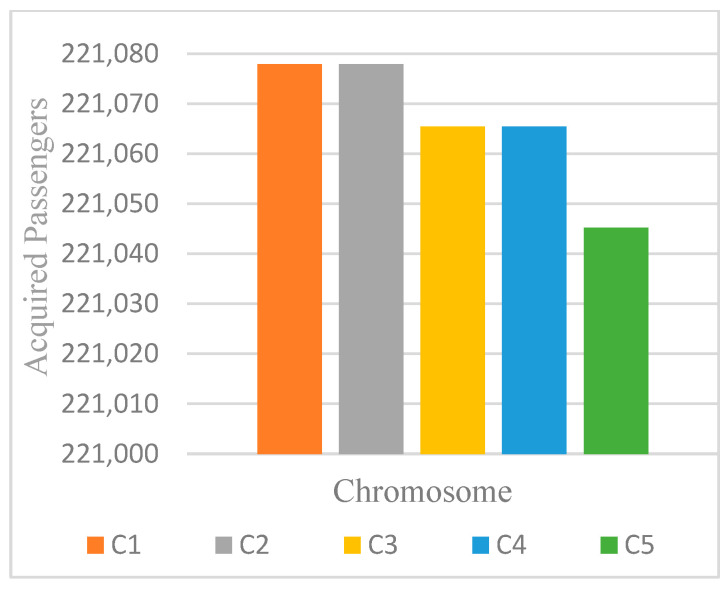
Passenger acquisition at iteration 3500 across chromosomes (C) for ES with seed 2.

Notably, the ES algorithm consistently outperformed others in both best and average results. Consequently, additional iterations were conducted with ES, extending to 3500 iterations, with results presented in Figure 13.

**Figure 14:**
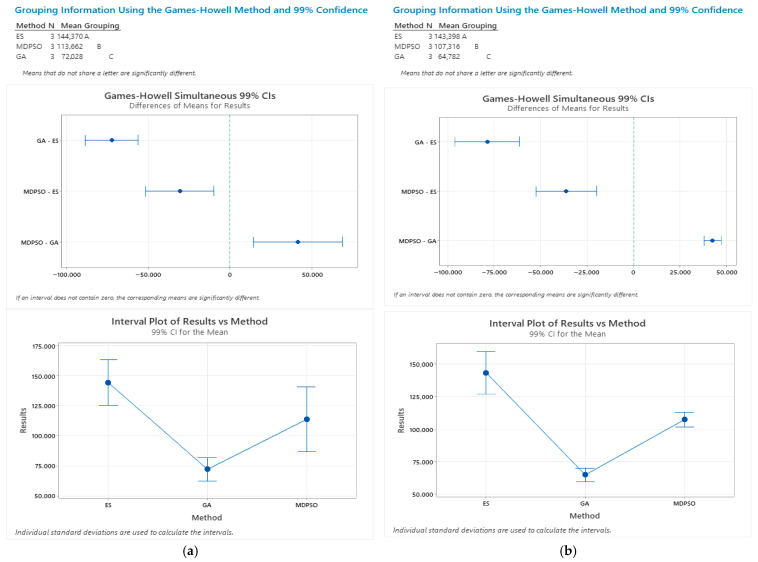
(**a**) One-way ANOVA and Games–Howell test results for the best outcomes of the three metaheuristic algorithms. (**b**) One-way ANOVA and Games–Howell test results for the average outcomes of the three metaheuristic algorithms.

To statistically assess the performance differences between the three metaheuristic algorithms (GA, MDPSO, and ES), a one-way ANOVA was conducted. This test is appropriate for comparing the mean performance metrics (best and average values) across multiple independent groups. Given the presence of unequal variances and differing sample sizes among groups, a Games–Howell post hoc test was employed for pairwise comparisons. A one-way ANOVA with unequal variances was conducted for both best and average results, yielding F-values of 514.16 and 1809.80, respectively, with *p*-values of 0.00 in both cases, indicating significant differences. Pairwise comparisons using the Games–Howell test confirmed that all intervals excluded zero, demonstrating statistically significant differences between the metaheuristic algorithms at α = 0.01 (Figure 14a,b).

Beyond numerical improvements, the results highlight important managerial and methodological insights regarding arrival–departure synchronization at hub airports. The comparative analysis demonstrates that solution quality and convergence speed differ substantially across metaheuristics, reflecting their distinct exploration–exploitation balances. While GA provides stable improvements, its reliance on crossover limits its ability to intensively refine tightly constrained schedules. MDPSO benefits from rapid information sharing among particles, yielding faster convergence, whereas ES consistently achieves superior solutions through mutation-driven local refinement. These findings confirm that synchronized optimization of arrivals and departures is critical for maximizing transfer flows and that evolutionary strategies are particularly well suited for large-scale, highly constrained airline scheduling problems.

## 7. Conclusions

Air transport continues to dominate long-distance mobility, yet the reliance on transfer flights makes the synchronization of arrivals and departures at hub airports a critical operational challenge. This study proposed and evaluated a metaheuristic-based framework for optimizing arrival–departure synchronization using Istanbul Airport—a central global hub—as a case study. Three algorithms were applied in parallel: Genetic Algorithms (GA), Modified Discrete Particle Swarm Optimization (MDPSO), and Evolutionary Strategies (ES).

The findings confirm that synchronization yields substantial improvements in transfer efficiency. While GA provided modest gains compared to the baseline, MDPSO and ES achieved significantly superior results. In particular, ES increased successful transfer passengers from approximately 65,000 under the original schedule to 147,000 (seed-2), reaching as high as 221,000 after extended iterations—representing more than a threefold improvement. These results demonstrate the capacity of evolutionary strategies to balance exploration and exploitation more effectively than alternative methods, thereby offering a robust approach for large-scale scheduling problems.

The proposed framework provides both theoretical and practical contributions. It advances the literature by being one of the few studies to jointly optimize arrivals and departures, a problem often overlooked in prior research focused primarily on flight or fleet scheduling. Practically, the results suggest that airlines and hub airports can substantially enhance passenger retention and connectivity by adopting metaheuristic optimization in scheduling decisions.

In addition to the performance gains observed, this study underscores the broader applicability of evolutionary metaheuristics for hub-based airline planning. The proposed framework demonstrates that significant improvements in passenger connectivity can be achieved without altering fleet size or network structure, solely through timetable synchronization. This is particularly valuable for congested hub airports where infrastructure expansion is limited. From a practical perspective, the results suggest that airlines can enhance competitiveness and passenger satisfaction through data-driven schedule optimization.

Future research should focus on integrating uncertainty, real-time disruptions, and hybrid metaheuristic approaches to further strengthen decision support for dynamic airline operations. In particular, extending the framework with advanced forecasting techniques and dynamic scheduling capabilities under real-world uncertainty—such as weather conditions or operational disruptions—would enhance its robustness. Additionally, testing hybrid metaheuristics can improve scalability and computational efficiency, thereby increasing the applicability of the proposed framework across diverse hub airports and airline networks worldwide.

## Figures and Tables

**Table 1:** Worldwide airline industry statistics [1,2].

Year	2019	2020	2021	2022	2023	2024 e	2025 f
Segment Passengers, million	4.560	1.779	2.304	3.452	4.426	4.779	4.988
Passenger Revenue, $bn	607	189	242	437	648	682	693
Cargo Revenue, $bn	101	140	210	206	139	149	142
Ancillary and Other Revenue, $bn	130	55	61	95	122	135	144
Net Profit, $bn	26.4	−137.7	−40.4	−3.5	37.3	32.4	36.0
Aircraft departures, million	37.5	19.7	24.2	29.5	35.5	37.4	38.3
Unique city pairs	20.886	16.218	16.259	20.424	21.736	>22.000	N/A

e: Estimates; f: Forecasts.

**Table 2:** Türkiye airline industry statistics [7,9].

		2003	2022	2023	2024	2024/2003 % Change
Passenger Traffic	Domestic	9,147,439	78,670,030	90,390,766	95,293,038	%941.7
	International	25,296,216	103,277,976	123,302,397	134,694,726	%432.5
	Transfer	0	385,838	443,412	236,847	-
	Total	34,443,655	182,333,844	214,136,575	230,224,611	%568.4
Airplane Traffic	Domestic	156,582	789,257	869,404	902,078	%476.1
	International	218,405	699,040	816,473	816,473	%273.8
	Transfer	154,218	394,889	485,453	521,724	%238.3
	Total	529,205	1,883,186	2,171,330	2,240,275	%323.3
Airplane Fleet Size		162	598	668	729	%350

**Table 3:** Passenger waiting-time preferences for connecting flights.

Waiting Time (h)	Waiting Rate (%)
0–1	0
1–3	100
3–5	50
5–7	20
7–10	10
10+	0

**Table 4:** Estimated Passenger Potential for Origin–Destination Travel: An Illustrative Example.

From	To	Average (Passengers)
A	X	15
A	Y	20
A	Z	25
…	…	…
Z	A	20
Z	B	15
Z	C	10

**Table 5:** Weekly Flight Schedule (Illustrative Example).

Point	Arrival in Hub-Airport	Departure from Hub-Airport
A	Monday (06:05), Sat (12:20)	Mon (12:20)
B	Tue (12:10), Sat (14:15)	Tue (16:00)
C	Wed (12:25), Thu (18:30)	Thu (22:25), Fri (17:45)
…	…	…
X	Fri (21:50), Sat (13:15)	Sun (12:15)
Y	Mon (11:05)	Mon (13:10), Fri (17:55)
Z	Fri (22:05)	Tue (12:35), Sat (01:20)

*Mon: Monday* *; Tue: Tuesday* *; Wed: Wednesday* *; Thu: Thursday* *; Fri: Friday* *; Sat: Saturday* *; Sun: Sunday.*

**Table 6:** Chromosome Structure for Arrival Scheduling.

		Arrival in Hub-Airport (X-Axis)			
Day (Y-Axis)	Hour (Y-Axis)	1	2	…	a
Monday	00:00			…	
Monday	00:05				
Monday	00:10				
…	…	…	…	…	…
Sunday	23:45				
Sunday	23:50				
Sunday	23:55			…	

**Table 7:** Chromosome Structure for Departure Scheduling.

		Departure from Hub-Airport (X-Axis)			
Day (Y-Axis)	Hour (Y-Axis)	1	2	…	d
Monday	00:00				
Monday	00:05				
Monday	00:10				
…	…	…	…	…	…
Sunday	23:45				
Sunday	23:50				
Sunday	23:55			…	

**Table 8:** Construction Stages.

Stages	Terminal (m^2^)	Annual Passenger Capacity (Million)	Runways
First Stage	1,440,000	90	5
Second stage			2
Third Stage	960,000	60	
Fourth stage *	800,000	50	1
**Total**	**3,200,000**	**200**	**8**

* (2 Satellite terminals).

**Table 9:** Runways.

Runways			
Direction	Length (m)	Width (m)	Surface
**16L/34R**	3750	45	Asphalt
**16R/34L**	3750	60	Asphalt
**17L/35R**	4100	60	Asphalt
**17R/35L**	4100	45	Asphalt
**18/36**	3060 *	45	A&C **

* Expandable to 3750 m, ** Asphalt & Concrete.

**Table 10:** Assumed demand between origin–destination pairs utilized in the mathematical model.

1. Point	2. Point	Dem.	1. Point	2. Point	Dem.	1. Point	2. Point	Dem.	1. Point	2. Point	Dem.	1. Point	2. Point	Dem.
ORD	IAD	19	IAD	ORD	11	LAX	ORD	17	JFK	ORD	7	YYZ	ORD	23
ORD	LAX	19	IAD	LAX	17	LAX	IAD	15	JFK	IAD	9	YYZ	IAD	15
ORD	JFK	11	IAD	JFK	19	LAX	JFK	5	JFK	LAX	11	YYZ	LAX	9
ORD	YYZ	27	IAD	YYZ	15	LAX	YYZ	4	JFK	YYZ	15	YYZ	JFK	10

*Dem: Demand (transfer passengers). Points indicate arrival and departure locations and represent different airports.*

**Table 11:** Optimized Schedule of Arrival and Departure Flights.

Flight Point	Departure Flight Time	Arrival Flight Time
ORD	5–10 min	10–15 min
IAD	10–15 min	5–10 min
LAX	10–15 min	5–10 min
JFK	15–20 min	0–5 min
YYZ	15–20 min	0–5 min

**Table 12:** Summary of Total Scheduled Arrivals and Departures.

	0–5 min	5–10 min	10–15 min	15–20 min	20–25 min
**Departure Flights**	0	1	2	2	0
**Arrival Flights**	2	2	1	0	0

**Table 13:** Number of Passengers Gained Through Synchronization.

1. Point	2. Point	Dem.	A.P.	1. Point	2. Point	Dem.	A.P.	1. Point	2. Point	Dem.	A.P.	1. Point	2. Point	Dem.	A.P.
ORD	IAD	19	0.0	IAD	LAX	17	17.0	LAX	JFK	5	2.5	JFK	YYZ	15	3
ORD	LAX	19	0.0	IAD	JFK	19	9.5	LAX	YYZ	4	2.0	YYZ	ORD	23	23
ORD	JFK	11	11.0	IAD	YYZ	15	7.5	JFK	ORD	7	7.0	YYZ	IAD	15	7.5
ORD	YYZ	27	27.0	LAX	ORD	17	0.0	JFK	IAD	9	4.5	YYZ	LAX	9	4.5
IAD	ORD	11	0.0	LAX	IAD	15	15.0	JFK	LAX	11	5.5	YYZ	JFK	10	2

*Points indicate arrival and departure Airports. Dem: Demand A.P: Acquired Passenger.*

**Table 14:** Comparative Performance of Genetic Algorithm Variants in Transfer Passenger Optimization.

GA Variant	Average of Best Values	Average of Avg. Values	Average of Worst Values
One-Point Crossover	75,693	63,619	57,620
Two-Point Crossover	69,270	62,956	56,907
Three-Point Crossover	79,372	64,632	57,394

**Table 15:** Transfer passenger acquisition results using Genetic Algorithm with three-point crossover.

Iter #	Seed1-Best	Seed1-Avg.	Seed2-Best	Seed2-Avg.	Seed3-Best	Seed3-Avg.	Seed4-Best	Seed4-Avg.	Seed5-Best	Seed5-Avg.	OP
0	72,367	55,994	67,171	53,956	68,460	50,387	67,911	53,268	68,335	56,547	65,659
30	72,372	65,871	67,173	62,490	71,314	62,363	117,105	70,920	68,901	61,520	
50	72,372	65,871	67,173	62,490	71,314	62,363	**117,105**	70,920	68,901	61,520	

*Avg.Best* = 79,372; *Avg.Avg* = 64,632; *Avg.Worst* = 57,394; *OP: Original Plan; Iter #: Iteration Number.*

**Table 16:** Parameter test results for ES and MDPSO.

	ES			MDPSO		
	Number of Chromosomes					
Genes	5	10	15	5	10	15
**100**	107,297	96,947	89,846	101,042	84,549	83,255
**300**	110,980 *	105,366	10,1233	104,783 **	88,052	90,838
**500**	108,484	106,672	99,814	99,688	95,661	95,167

*, ** The optimal performance of both ES and MDPSO is achieved with 300 *mutated genes and a population size of* 5.

**Table 17:** Performance of Genetic Algorithm with Three-Point Crossover Across Seeds.

Iter #	Seed1-Best	Seed1-Avg.	Seed1-Worst	Seed2-Best	Seed2-Avg.	Seed-Worst	Seed3-Best	Seed3-Avg.	Seed3-Worst	OP
20	73,107	62,157	56,737	72,888	65,267	61,740	70,089	65,327	60,224	65,659
40	73,107	62,157	56,737	72,888	65,267	61,740	70,089	65,327	60,224	
60	73,107	62,157	56,737	72,888	65,267	61,740	70,089	65,327	60,224	
80	73,107	62,157	56,737	72,888	65,267	61,740	70,089	65,327	60,224	
100	73,107 *	63,753	60,675	72,888	65,267	61,740	70,089	65,327	60,224	

*OP: Original Plan*; *Iter #: Iteration number*; * indicates the best solution obtained.

**Table 18:** Performance Results of MDPSO Across Seeds.

Iter #	Seed-1 Best	Seed-1 Average	Seed-1 Worst	Seed-2 Best	Seed-2 Average	Seed-2 Worst	Seed-3 Best	Seed-3 Average	Seed-3 Worst	OP
40	78,447	73,642	65,807	82,320	77,192	72,048	82,737	77,259	71,337	65,659
80	86,775	82,951	77,927	93,934	86,229	82,559	91,915	88,417	85,514	
120	96,136	92,593	86,558	97,902	93,266	87,661	102,883	95,566	91,024	
160	108,931	102,020	92,473	104,362	99,758	93,012	113,322	103,803	95,758	
200	114,197	106,247	100,593	108,696	107,466	106,248	118,092 *	108,236	98,987	

OP: Original Plan; Iter #: Iteration number; * indicates the best solution obtained.

**Table 19:** Best, average, and worst results across three seeds for ES.

Iter #	Seed-1 Best	Seed-1 Average	Seed-1 Worst	Seed-2 Best	Seed-2 Average	Seed-2 Worst	Seed-3 Best	Seed-3 Average	Seed-3 Worst	OP
40	99,987	99,278	98,532	105,508	102,960	101,172	88,481	881,72	87,918	65,659
80	11,4001	112,652	111,796	123,808	123,504	123,250	106,834	106,341	10,5803	
120	126,137	125,615	125,307	136,197	133,189	131,507	122,682	121,497	120,952	
160	136,914	135,424	134,682	143,861	141,830	141,113	131,967	130,291	129,783	
200	144,520	143,785	143,463	147,596 *	146,019	145,281	140,993	140,389	139,991	

OP: Original Plan; Iter #: Iteration number; * indicates the best solution obtained.

## Data Availability

Data available in a publicly accessible repository.
